# Dietary Diversity and All-Cause and Cause-Specific Mortality in Japanese Community-Dwelling Older Adults

**DOI:** 10.3390/nu12041052

**Published:** 2020-04-10

**Authors:** Rei Otsuka, Chikako Tange, Yukiko Nishita, Yuki Kato, Makiko Tomida, Tomoko Imai, Fujiko Ando, Hiroshi Shimokata

**Affiliations:** 1 Section of NILS-LSA, National Center for Geriatrics and Gerontology, Aichi 474-8511, Japan; tange@ncgg.go.jp (C.T.); kyuki@asu.aasa.ac.jp (Y.K.); tomida@ncgg.go.jp (M.T.); toimai@dwc.doshisha.ac.jp (T.I.); fujikoa@asu.aasa.ac.jp (F.A.); simokata@nuas.ac.jp (H.S.); 2 Department of Epidemiology of Aging, National Center for Geriatrics and Gerontology, Aichi 474-8511, Japan; nishita@ncgg.go.jp; 3 Faculty of Health and Medical Sciences, Aichi Shukutoku University, Aichi 480-1197, Japan; 4 Faculty of Human Life and Science, Doshisha Women’s College of Liberal Arts, Kyoto 602-0893, Japan; 5 Graduate School of Nutritional Sciences, Nagoya University of Arts and Sciences, Aichi 470-0196, Japan

**Keywords:** dietary diversity, all-cause mortality, cancer mortality, Japanese, older people

## Abstract

We examined associations between dietary diversity and all-cause and cause-specific mortality in 386 men and 413 women (age range, 60–79 years at baseline) who took part in the National Institute for Longevity Sciences-Longitudinal Study of Aging study from 1997 to 2000. Dietary intake was assessed using three-day dietary records and photographs. The Quantitative Index for Dietary Diversity was used to determine the dietary diversity among thirteen food groups. Dietary diversity score and each food intake were examined by sex-stratified tertiles, and hazard ratios (HR) were calculated to compare the risk for all-cause and cause-specific deaths across tertiles, after controlling for age, sex, body mass index, alcohol intake, smoking status, education, physical activity, and disease history. During a mean follow-up of 15.7 years, 289 subjects (36.2%) died. Compared to the subjects in the lowest tertile, the multivariate-adjusted HR for all-cause and cancer mortality was 0.69 (95% confidence interval (CI): 0.51–0.94) and 0.57 (95% CI: 0.33–0.98), respectively (trend *p* < 0.05), in subjects in the highest tertile of dietary diversity. There were no significant associations between dietary diversity score and death from cardiovascular or cerebrovascular disease. Eating a variety of foods might contribute to longevity in older Japanese community dwellers.

## 1. Introduction

Extending the healthy lifespan is important in today’s rapidly aging society, and dietary diversity has been shown to be positively associated with a healthy life expectancy, even after controlling for gross domestic product [1].

An imbalanced diet or consuming a low variety of foods might lead to multiple nutritional deficiencies. Compared to younger people, older people have a higher risk for being nutritionally vulnerable as well as having lower dietary diversity, due to loss of a spouse, loss of chewing ability, low appetite, or eating poorly due to economic considerations [2,3]. Previously, we reported that dietary diversity among older Japanese subjects was positively associated with a higher level functional capacity [4] and cognitive function [3]. Based on these reports, we considered that eating a variety of foods might be one of the best population approaches to prevent functional decline and dementia. However, the longitudinal association of dietary diversity on health outcomes in later life, such as lifespan, is unknown.

Epidemiological studies in Western countries indicate that dietary diversity is closely associated with decreases in mortality [5] and cancer [6]. The primary underlying mechanism for this decreased mortality is that a higher dietary diversity is associated with a more favorable nutritional intake [4]. On the other hand, dietary diversity was reported to be positively associated with obesity [7]. As someone with a higher dietary diversity score might tend to eat more food, eating a variety of foods might also be a risk factor for lifestyle-related diseases. However, older adults sometimes experience a loss of appetite, and a poor appetite was shown to be associated with lower food and nutrient intakes, and was a risk factor for mortality in older Taiwanese subjects [8]. Therefore, maintaining dietary diversity among the elderly to potentially increase longevity must also involve a nutritionally rich diet. A large Japanese prospective study reported that consuming a greater diversity of foods decreased total mortality and cardiovascular risk for women but not for men [9]. However, that study included middle-aged subjects, and the age range of the subjects was large (aged 45 to 74 years). More research focusing on dietary diversity and mortality among older subjects is needed to understand the relationship between dietary diversity and lifespan.

This study examined the associations of dietary diversity, calculated by dietary records and photographs, with all-cause and cause-specific mortality, in community-dwelling older Japanese subjects.

## 2. Materials and Methods

### 2.1. Baseline Survey Participants

Data for this survey were taken from the National Institute for Longevity Sciences–Longitudinal Study of Aging (NILS–LSA). This study used detailed questionnaires and medical checkups, anthropometric measurements, physical fitness tests, and nutritional examinations to assess the normal aging process, over time. Participants in the NILS–LSA were randomly selected age- and sex-stratified individuals from a pool of community-dwelling residents in the National Center for Geriatrics and Gerontology neighborhood areas of the Obu City and Higashiura Town in the Aichi Prefecture. The first wave of the NILS–LSA took place from November 1997 to April 2000 and included 2267 participants (1139 men, 1128 women; age range, 40–79 years). Subjects were followed up every 2 years from the second wave of the study (April 2000–May 2002) to the seventh wave of the study (July 2010–July 2012).

Details of the NILS–LSA study were reported previously [10]. The Committee of Ethics of Human Research of the National Center for Geriatrics and Gerontology approved the study protocol (No. 899-5). Written, informed consent was obtained from all participants.

### 2.2. Follow-Up and Study Subjects

Vital status was determined using residence records, and local government data were used to determine if the participants had moved away. Cause of death was based on the National Vital statistics records available till the end of December 2017. Codes from the tenth International Classification of Diseases were used to identify the underlying cause of death [11]. Deaths were confirmed by computer matching of data from the National Vital Statistics records, using the following key codes—resident areas, sex, and dates of birth and death.

Among the total of 2267 participants at baseline, 1133 aged ≥ 60 years were included in the present study. Exclusion criteria were (1) those without completed dietary records (*n* = 43); (2) those with current/past cancer (*n* = 64); (3) those with current/past cerebrovascular disease (*n* = 48); (4) those with current/past heart disease (*n* = 158); and (5) those with missing data for controlled variables (*n* = 21). Subjects with cancer, cerebrovascular disease, or cardiovascular disease were excluded because these diseases are the three major causes of death among the Japanese, and as such, these subjects would have a higher mortality risk at baseline. A total of 386 men and 413 women aged 60 to 79 years at baseline were included in this analysis.

### 2.3. Dietary Assessments

After participation in the baseline study, subjects completed a 3-day dietary record for assessment of dietary intake. The dietary record was completed over 3 continuous days (2 weekdays and 1 weekend day) [12]. Subjects completed the dietary record at home, and most returned it within 1 month. Food was weighed separately on a 1-kg kitchen scale (Sekisui Jushi, Tokyo, Japan) before being cooked, or portion sizes were estimated. Subjects used a disposable camera (27 shots; Fuji Film, Tokyo, Japan) to take photos of meals before and after eating. Dietitians used these photos to complete missing information on the dietary record. Any discrepancies or needs for additional information were obtained via telephone calls to subjects. Averages for food and nutrient intakes (including alcohol intake) over the 3 days were calculated according to the Standard Tables of Food Composition in Japan 2010, and other sources [12,13].

### 2.4. Dietary Diversity Score

Dietary diversity was determined using the Quantitative Index for Dietary Diversity (QUANTIDD) [14].

(1) $$\mathrm{QUANTIDD}=\frac{1-\sum_{j}^{n}prop{\left(j\right)}^{2}}{1-\frac{1}{n}}$$

where *prop(j)* is the proportion of food group(s) *j* that contribute to total energy or nutrient intake, *n* is the number of food groups, and *j* = 1,2,…,n.

QUANTIDD is calculated by the proportion of foods that contribute to total energy, or the amount of foods and the number of food groups. The index ranges from 0 to 1. Lower scores indicate an unbalanced diet, and higher scores indicate an equal distribution of each food group.

There are 18 food groups in the 2010 Japanese food composition table [13]—grains, potatoes and starches, sugars and sweeteners, pulses, nuts and seeds, vegetables, fruits, mushrooms, algae, fish and shellfish, meat, eggs, dairy products, fats and oils, confectionaries, beverages, seasoning and spices, and prepared and processed foods. In this study, we calculated the score based on the amount (grams) of 13 food groups that included cereals (grains), potatoes (potatoes and starches), beans (pulses), nuts and seeds, non-green yellow vegetables, green yellow vegetables, fruits, mushrooms, seaweed (algae), fish and shellfish, meats, eggs, milk and dairy products (dairy products), excluding sugars and sweeteners, fats and oils, confectionaries, beverages, seasoning and spices, and prepared and processed foods. We selected only solid foods, excluding beverages (liquid foods) and seasonings, including sugars. The reason for excluding beverages was that the beverage category in the Japanese food composition table include alcohol, tea, and fruit-flavored and colored drinks, but not fresh fruit juice or milk. We excluded beverages as a component of the dietary diversity score to avoid difficulties in measuring beverages that contain a mix of alcohol, tea, and fruit-flavored or colored drinks. In sensitivity analyses, we calculated the score based on the amount (grams) of 18 food groups. There was no positive association between dietary diversity score and beverages with or without alcohol, as well as between dietary diversity score and mortality.

### 2.5. Other Baseline Measurements

Education years (≤9, 10–12, or ≥13), smoking status (yes or no), and history of diseases were collected using self-reported questionnaires, and data were confirmed by medical doctors or trained staff. Data on past and current dyslipidemia, hypertension, and diabetes mellitus was divided into 4 categories—(1) none; (2) on medication; (3) previously medicated; and (4) not treated. These diseases were then re-categorized into 2 categories (yes or no; yes included on medication, previously medicated, and not treated, and no included none). Physical activity was assessed using the metabolic equivalents score, which represents a multiple of the resting metabolic rate. Trained interviewers used a semi-quantitative assessment to determine levels of regular physical activity during leisure time and on the job, as well as sleeping hours [15]. Body mass index (BMI) was calculated as weight in kilograms divided by the square of the height in meters.

### 2.6. Statistical Analysis

Sex-stratified tertiles of food intakes and dietary diversity were used as explanatory variables. The Cox proportional hazards model was used to assess relationships between food intakes and dietary diversity with all-cause and cause-specific mortality. Findings were expressed using hazard ratios (HRs) and 95% confidence intervals (CIs). Outcomes were calculated by tertiles. Follow-up time (years) was calculated by the number of days that had elapsed from the baseline study. The primary endpoint was time at death or the latest date of the adjudicated follow-up data until the end of December 2017. The confounding variables included sex, baseline age, BMI [16], smoking status [17], alcohol drinking [18], physical activity [19], education [20], history of hypertension, dyslipidemia, and diabetes mellitus [21,22]. Energy intake and protein intake did not show any association with mortality risk in our pre-analysis, and our goal was to show that how one eats is associated with longevity as opposed to how much one eats. Thus, we did not account for any nutritional intake other than dietary diversity.

The χ2 test was used to determine differences in proportions by tertiles of dietary diversity, and one-way analysis of variance was used for comparisons between continuous variables according to the tertiles of dietary diversity (Table 1). A sex-adjusted linear regression model was used to examine the association between dietary and nutritional intakes and dietary diversity score (Table 2). All dietary or nutritional intakes were entered into a single model.

All *p* values were two-sided, and a *p* value < 0.05 was considered to be significant. Statistical analyses were conducted using the Statistical Analysis System software version 9.3 (SAS Institute, Cary, NC, USA).

## 3. Results

From the baseline survey to December 2017, 289 subjects (36.2%) died. The mean (standard deviation (SD)) follow up was 15.7 (4.7) years. Follow-up of survivors was 17.9 (2.9) years, and that of the non-survivors was 11.9 (4.6) years. There were 289 deaths due to cancer (*n* = 92; 31.8%), cardiovascular disease (*n* = 38; 13.1%), and stroke (*n* = 31; 10.7%).

Table 1 shows the baseline characteristics according to the tertiles of dietary diversity score. Subjects with higher dietary diversity drank significantly more alcohol, had a significantly higher education level, and a lower prevalence of smoking. Table 2 shows the baseline dietary and nutritional intakes according to the tertiles of dietary diversity score. Subjects with higher dietary diversity scores ate significantly less cereal and significantly more of the other 12 food groups. Among cereals, consumption of whole grains including oats, whole grain corn or wheat, and brown rice, was low, although intakes of these foods were higher among subjects in the higher tertiles of dietary diversity score. In terms of meat intake, consumption of red meat, including beef and pork, was not associated with the dietary diversity score. For nutrient intake, subjects with higher dietary diversity scores consumed significantly more energy, protein, and the other 8 nutritional intakes, including sodium. In terms of protein and fat intake, both plant and animal protein, and saturated-, *n*-3, or *n*-6 polyunsaturated fatty acids intake also increased with the dietary diversity score.

Table 3 shows the multivariate-adjusted HRs for all-cause mortality, according to the tertiles for each food intake. None of the food group intakes used to calculate the dietary diversity score was positively associated with the risk for all-cause mortality (*p* for trend > 0.05).

Table 4 shows the multivariate-adjusted HRs for all-cause and cause-specific mortality, according to the tertiles of dietary diversity score. The HR for all-cause mortality of the highest tertile of dietary diversity score was 0.69 (95% CI: 0.51–0.94), compared to the lowest tertile of dietary diversity score (trend *p* = 0.0186). For cause-specific mortality, the HR for cancer mortality of the highest tertile of dietary diversity score was 0.57 (95% CI: 0.33–0.98) compared to the lowest tertile of dietary diversity score (trend *p* = 0.0466). Dietary diversity score was not associated with cardiovascular or cerebrovascular mortality.

In additional analyses, no statistically positive associations were found between food intake and cancer mortality (Table S1), cardiovascular mortality (Table S2), or cerebrovascular mortality (Table S3) (*p* for trend > 0.05).

## 4. Discussion

These results suggest a beneficial effect of higher dietary diversity on all-cause and cancer-specific mortality in older Japanese subjects. In addition, none of the individual food intakes in the dietary diversity score was associated with all-cause or cause-specific mortality. This finding suggests that a balanced diet that contains a variety of foods, as opposed to specific foods, might have a beneficial effect on life expectancy.

Previous reports indicated that dietary diversity was closely associated with decreases in mortality [5,9,23] and cancer [6]. However, most of these studies defined the dietary diversity score based on only 5 or 6 foods groups. Only one large prospective study among the Japanese defined this score based on the 133 food items, but they did not study the amount of food eaten [9]. In our study, we evaluated dietary diversity based on 13 foods groups and the amount eaten, and showed that dietary diversity was positively associated with longevity. The standard Japanese diet is characterized by its use of a wide variety of seasonal ingredients, as well as abundant aquaculture products [24], but, according to the Japanese food composition table, daily meals can be classified into just 13 food groups [13]. Even when considering the amount of food eaten, high dietary diversity based on 13 foods groups was positively associated with longevity. In this study, we defined dietary diversity as the proportion of foods that contribute to the total amount of foods. We selected 13 of 18 food groups from the Japanese food composition table, as we did not want to consider intake of unfavorable foods. This methodology was chosen both to keep our methods simpler and to allow us to come up with understandable public health nutrition messages regarding food diversity that are important to the elderly. In fact, previous studies recommended eating whole grains for health [25], and red meat intake was associated with colon cancer [26]. In sub-analyses, we analyzed the association between whole grains, alcohol or red meat intake, and all-cause or cause-specific mortality, but no positive associations were found. The reason for this lack of association might be the small intake of whole grains and alcohol and lower intake of red meat among Japanese subjects, as compared to Western subjects [25,26].

There are two main dietary mechanisms that contribute to longer lifespan that should be considered. One is metabolic mechanisms, as subjects with higher dietary diversity ate more animal/plant protein, fat including saturated or polyunsaturated fatty acid, minerals, and vitamins. The dietary reference intakes (DRI) for the Japanese propose reference values of desirable dietary intake of energy and nutrients for Japanese people, to maintain and promote their health [27]. For 18 nutrients including protein, sodium, calcium, magnesium, iron, zinc, vitamin A and vitamin C (shown in Table 2), sex- and age-stratified estimated average requirements were defined to ensure adequate intakes of these foods. For fats and saturated fatty acids, tentative dietary goals were developed to help prevent lifestyle-related diseases (e.g., recommended fat intake of 20–30% of energy in all sex and age groups). In this study, we sub-analyzed the prevalence of achieving the DRI score (for each nutrient) for each tertile of the dietary diversity score, and showed that subjects with a higher dietary diversity score tended to have more desirable nutritional intake, that is, they tended to consume more nutritionally rich food, based on the Japanese DRI. Therefore, we confirmed that the higher dietary diversity calculated by QUANTIDD was associated with a more nutritionally rich diet. In addition, subjects with a higher dietary diversity tended to consume more dietary fiber (Table 2). However, we were unable to assess the microbiome of subjects, and this variable has been suggested to contribute to human longevity, based on the complex interaction between an organism and its environment [28,29]. These recommended nutrients, such as minerals and vitamins, also have anti-inflammatory and antioxidant effects, and a combination of several kinds of nutrients might extend the lifespan. In fact, most studies focused on healthy eating [30] show that diets with lots of vegetables, fruits, nuts, fish, and grained cereals reduce mortality. A meta-analysis indicated that healthy dietary patterns were negatively associated with the risk for all-cause, cancer, and cardiovascular mortality [31].

The second mechanism regarding dietary behavior, that is, eating a variety of foods daily, requires health consciousness and healthy activities, such as shopping, cooking, and planning a menu at least three times per day. These intentional and complex instrumental activities require multiple cognitive processes [6]. Although we could not consider cognitive function, frailty or disability that occurred before death in our subjects, eating a variety of foods daily, along with the associated activities might be effective for the maintenance of functional capacity [4] or physical performance [32]. Therefore, it is possible that eating or preparing a variety of foods might have a favorable effect on longer life based on the higher functional capacity required to complete associated activities.

In this study, we focused on older subjects because these subjects were suspected to have low dietary diversity, as compared to younger subjects, due to a higher risk of being nutritionally vulnerable [2,3]. A Taiwanese study of an older population reported that a greater dietary diversity was associated with lower emergency and hospitalization utilization and expenditures, but not with lower use of ambulatory services [33]. In addition, a recent Taiwanese study reported that dietary diversity offset the adverse mortality risk among older indigenous Taiwanese [23]. On the other hand, dietary diversity did not offset the mortality risk associated with hyperhomocysteinemia in older adults with diabetes [34]. These results might mean that dietary diversity is effective for longer healthy lifespans, but is less effective for patients with metabolic diseases. In addition, older subjects with frailty or malnutrition might have nutritional disadvantages [35]. A higher dietary variety was reported to be associated with better nutritional status in frail elderly people [36]. So, even in the frail elderly, eating a variety of foods might be one of the best population approaches to encourage public nutrition because older people only need to be aware of eating some types of food every day.

There were no positive associations between baseline dietary diversity and cardiovascular or cerebrovascular mortality. This lack of finding might be due to the small sample size in this study. We excluded subjects with current/past cancer (*n* = 64), cerebrovascular disease (*n* = 48), or cardiovascular disease (*n* = 158). As a result, 13.1% (*n* = 38) or 10.7% (*n* = 31) died due to a cardiovascular or cerebrovascular disease, among 289 deaths. In fact, effect size (Cramer’s V) of these analyses were 0.02 or 0.07, respectively, which was small. Larger prospective studies are needed to understand the association between dietary diversity and cause-specific mortality among Japanese subjects.

Several limitations to the present study warrant consideration. First, we assessed dietary diversity from a single nutritional assessment at baseline. Food intake is easily changeable and affected by various factors associated with aging. Second, Japanese intake of meat and dairy products is considered low by global standards [24,37]. In addition, Japanese individuals eat a wider diversity of foods [5]. Therefore, the present findings might not be generalization of Western populations who consume larger amounts of meat and a lower variety of foods. Third, we could not determine health status or health-related lifestyle factors, including physical activity, during the follow-up period. Sarcopenia, frailty, and disability might have occurred before death in some subjects. In addition, we could only analyze the main causes of deaths, and thus, were unable to determine the death rates for specific types of cancer. Effect size of the prevalence of all-cause mortality in this study was 0.10 (Cramer’s V), which was small. Thus, our results might underestimate the impact on death and on health factors associated with different types of cancer. 

The strengths of our study were—(1) the long follow-up period (mean, 16 years); (2) older subjects who were randomly recruited from age- and sex-stratified community dwellers; and (3) dietary diversity evaluated on the basis of 13 foods groups and the amount eaten from the three-day dietary records and photographs. This type of dietary assessment could be associated with less recall bias than that associated with dietary recall or self-reported questionnaires. Therefore, the results of our study provided more convincing evidence that dietary diversity might lead to longevity among community dwellers.

## 5. Conclusions

Eating a variety of foods might be recommended for longevity among Japanese community dwellers.

## Figures and Tables

**Table 1:** Baseline characteristics according to the tertiles of dietary diversity score.

	Tertiles of Dietary Diversity Score ^1^							
	Lowest Tertile		Middle Tertile		Highest Tertile		*P* ^2^	Trend *p*
	*n* = 265		*n* = 267		*n* = 267			
	Mean	SD	Mean	SD	Mean	SD		
Dietary diversity score	0.81	0.05	0.89	0.01	0.92	0.01	<0.0001	<0.0001
Median	0.83		0.89		0.92			
Range (min-max) in men	0.614–0.849		0.850–0.895		0.896–0.957			
Range (min-max) in women	0.631–0.880		0.881–0.907		0.908–0.964			
Men (%)	48.3		48.3		48.3		0.99	
Age (years)	68.4	5.6	68.0	5.4	67.9	5.3	0.58	0.31
Body Mass Index (kg/m^2^)	22.6	3.3	22.9	3.0	23.0	3.0	0.34	0.14
Alcohol (ml/day)	6.0	13.6	7.0	11.6	9.4	16.6	0.02	0.01
Physical activity(METS*hr/day)	34.7	4.4	34.9	3.8	34.6	3.6	0.65	0.75
Current smoker (%)	23.4		18.7		12.7		<0.001	
Education								
≤9 years (%)	61.1		47.6		37.5		<0.001	
10–12 years (%)	28.7		38.6		40.5			
≥13 years (%)	10.2		13.9		22.1			
Employment								
Unemployed (%)	57.0		71.5		69.6		<0.001	
Regular employment (%)	25.7		18.7		19.1			
Non-regular employment (%)	17.4		9.7		11.2			
Diseases								
Hypertension (%)	34.7		37.8		29.2		0.10	
Dyslipidemia (%)	18.1		17.2		23.2		0.17	
Diabetes (%)	9.4		8.6		12.0		0.40	

SD—Standard Deviation, METS*hr—Metabolic Equivalents Score times hour. ^1^ Dietary diversity were divided into sex-stratified tertiles. ^2^ One-way analysis of variance was used for continuous variables; χ^2^ test was used for the categorical variables.

**Table 2:** Baseline dietary and nutritional intakes according to the tertiles of dietary diversity score and association between dietary and nutritional intakes and dietary diversity score by multivariate linear regression ^1.^

	Tertiles of Dietary Diversity Score						Dietary Diversity Score	
	Lowest Tertile		Middle Tertile		Highest Tertile			
	*n* = 265		*n* = 267		*n* = 267		*n* = 799	
	Mean	SD	Mean	SD	Mean	SD	β *^2^*	*p*
Food intake								
Cereals (g/day)	558.9	158.9	475.2	121.3	392.5	101.0	−0.598	<0.001
Whole grains (g/day)	2.6	18.8	4.8	33.3	7.1	31.6	−0.013	0.507
Potatoes (g/day)	39.8	35.1	53.6	40.9	66.1	47.0	0.171	<0.001
Beans (g/day)	55.6	40.9	69.7	50.2	93.3	58.4	0.158	<0.001
Nuts and seeds (g/day)	2.8	6.5	4.8	8.2	5.4	10.4	0.040	0.032
Non-green yellow vegetables (g/day)	154.3	79.9	185.5	81.2	206.0	83.5	0.156	<0.001
Green yellow vegetables (g/day)	94.0	66.1	127.5	76.2	157.0	71.1	0.163	<0.001
Fruits (g/day)	131.0	136.8	183.2	121.8	201.4	105.3	0.143	<0.001
Mushrooms (g/day)	11.0	13.7	14.1	13.2	18.7	17.8	0.074	0.001
Seaweed (g/day)	15.1	26.0	14.9	14.1	19.5	19.3	0.081	<0.001
Fish and shellfish (g/day)	90.2	55.9	97.3	45.1	108.9	50.4	0.137	<0.001
Meats (g/day)	46.9	29.4	51.5	29.3	56.2	31.8	0.139	<0.001
Red meat (g/day)	24.5	22.1	25.2	22.2	28.2	23.3	−0.014	0.57
Eggs (g/day)	40.9	25.8	45.4	25.1	51.0	24.0	0.127	<0.001
Milk and dairy products (g/day)	100.6	121.6	181.9	136.0	213.9	111.5	0.206	<0.001
Nutritional intake								
Energy (kcal/day)	1986.8	429.8	2087.3	418.4	2112.9	405.5	−0.527	<0.001
Protein (g/day)	71.6	17.0	79.1	16.8	86.2	16.9	-	-
Animal protein (g/day)	34.8	13.4	40.9	12.0	46.1	12.0	0.361	<0.001
Plant protein (g/day)	36.8	8.0	38.2	8.2	40.0	8.9	−0.204	0.0014
Fat (g/day)	44.6	12.6	53.9	15.3	58.9	15.6	-	-
Saturated fatty acids (g/day)	12.1	4.3	15.3	5.4	16.6	4.9	0.194	<0.001
*n*-6 PUFA (g/day)	8.6	2.6	10.1	3.1	11.2	3.6	0.270	<0.001
*n*-3 PUFA (g/day)	2.1	0.9	2.4	1.0	2.7	1.0	−0.056	0.10
Sodium (mg/day)	4339.1	1234.0	4611.3	1301.0	4889.6	1186.8	0.0008	0.98
Calcium (mg/day)	516.3	190.1	676.2	212.3	795.1	221.6	0.182	<0.001
Magnesium (mg/day)	282.4	70.3	323.5	75.5	364.0	86.0	0.089	0.19
Iron (mg/day)	8.9	2.4	9.9	2.5	11.2	2.7	−0.037	0.46
Zinc (mg/day)	8.7	2.2	9.3	2.1	9.9	2.2	−0.093	0.042
Vitamin A (μg/day)	697.8	1060.2	841.9	827.0	878.0	775.4	0.034	0.22
Vitamin C (mg/day)	118.9	60.7	152.0	68.9	172.6	64.0	0.124	<0.001
Fiber (g/day)	15.2	4.3	17.7	4.2	28.2	23.3	0.405	<0.001

PUFA—Poly unsaturated fatty acids. ^1^ A sex-adjusted linear regression model was used. Independent variables were dietary or nutritional intakes. The dependent variable was dietary diversity score. ^2^ Standardized beta coefficients. All dietary or nutritional intakes were entered into a single model.

**Table 3:** Multivariate-adjusted hazard ratios ^1^ for all-cause mortality, according to the tertiles of each food intake.

	Tertiles of Each Food Intake			
	Lowest Tertile	Middle Tertile	Highest Tertile	*p*
Cereal				
Median (g/day)	350	441.8	603.3	
Number of each tertile	264	268	267	
Death *n*, %	102, 38.6%	85, 31.7%	102, 38.2%	
HR (95% CI)	1 (ref)	0.86 (0.63–1.18)	1.03 (0.76–1.39)	0.487
Potatoes				
Median (g/day)	15.7	43.8	86.7	
Number of each tertile	261	271	267	
Death *n*, %	95, 36.4%	108, 39.8%	86, 32.2%	
HR (95% CI)	1 (ref)	1.04 (0.78–1.39)	0.85 (0.62–1.16)	0.381
Beans				
Median (g/day)	25.8	61.7	117	
Number of each tertile	264	264	271	
Death *n*, %	101, 38.2%	99, 37.5%	89, 32.8%	
HR (95% CI)	1 (ref)	0.94 (0.70–1.26)	0.78 (0.57–1.05)	0.233
Nuts and seeds				
Median (g/day)	0	1.3	7.3	
Number of each tertile	265	256	278	
Death *n*, %	114, 43.0%	90, 35.1%	85, 30.5%	
HR (95% CI)	1 (ref)	0.95 (0.71–1.27)	0.78 (0.58–1.05)	0.247
Non-green yellow vegetables				
Median (g/day)	99.8	172.7	259.1	
Number of each tertile	265	267	267	
Death *n*, %	113, 42.6%	83, 31.0%	93, 34.8%	
HR (95% CI)	1 (ref)	0.79 (0.59–1.07)	1.03 (0.76–1.41)	0.181
Green yellow vegetables				
Median (g/day)	57	114.4	187.6	
Number of each tertile	265	267	267	
Death *n*, %	104, 39.2%	95, 35.5%	90, 33.7%	
HR (95% CI)	1 (ref)	0.94 (0.69–1.28)	1.09 (0.79–1.50)	0.638
Fruits				
Median (g/day)	61	148.5	276.7	
Number of each tertile	262	270	267	
Death *n*, %	105, 40.0%	89, 32.9%	95, 35.5%	
HR (95% CI)	1 (ref)	0.92 (0.68–1.25)	1.09 (0.80–1.48)	0.549
Mushrooms				
Median (g/day)	0	10	27.6	
Number of each tertile	259	271	269	
Death *n*, %	101, 39.0%	97, 35.7%	91, 33.8%	
HR (95% CI)	1 (ref)	0.95 (0.71–1.29)	0.97 (0.72–1.32)	0.952
Seaweed				
Median (g/day)	3.4	11.3	27.5	
Number of each tertile	265	267	267	
Death *n*, %	100, 37.7%	94, 35.2%	95, 35.5%	
HR (95% CI)	1 (ref)	0.78 (0.58–1.05)	0.90 (0.67–1.22)	0.258
Fish and shellfish				
Median (g/day)	54.1	89.5	143.3	
Number of each tertile	265	267	267	
Death *n*, %	103, 38.8%	89, 33.3%	97, 36.3%	
HR (95% CI)	1 (ref)	0.96 (0.71–1.29)	1.06 (0.79–1.44)	0.793
Meats				
Median (g/day)	21.7	48.3	80	
Number of each tertile	265	266	268	
Death *n*, %	108, 40.7%	98, 36.8%	83, 30.9%	
HR (95% CI)	1 (ref)	1.01 (0.76–1.36)	0.92 (0.68–1.26)	0.823
Eggs				
Median (g/day)	20	45	68.8	
Number of each tertile	264	267	268	
Death *n*, %	93, 35.2%	104, 38.9%	92, 34.3%	
HR (95% CI)	1 (ref)	1.17 (0.87–1.56)	1.09 (0.81–1.48)	0.589
Milk and dairy products				
Median (g/day)	13.3	155.7	285.3	
Number of each tertile	262	270	267	
Death *n*, %	105, 40.0%	98, 36.3%	86, 32.2%	
HR (95% CI)	1 (ref)	0.84 (0.63–1.13)	0.82 (0.60–1.13)	0.398

HR—Hazard ratio, CI—Confidence Interval, ref—reference. ^1^ HR adjusted for age, sex, body mass index, education, smoking status, alcohol intake, physical activity and history of hypertension, dyslipidemia, and diabetes mellitus. All food intakes were entered into a single model.

**Table 4:** Multivariate-adjusted hazard ratios ^1^ for all-cause and cause-specific mortality, according to the tertiles of dietary diversity score.

	Tertiles of Dietary Diversity Score			
	Lowest Tertile	Middle Tertile	Highest Tertile	Trend *p*
	*n* = 265	*n* = 267	*n* = 267	
All-cause mortality				
Death *n*, %	114, 43.0%	90, 33.7%	85, 31.8%	
HR (95% CI)	1 (ref)	0.76 (0.57–1.01)	0.69 (0.51–0.94)	0.0186
Cancer mortality				
Death *n*, %	39, 14.7%	28, 10.4%	25, 9.3%	
HR (95% CI)	1 (ref)	0.75 (0.45–1.24)	0.57 (0.33–0.98)	0.0466
Cardiovascular mortality				
Death *n*, %	13, 4.9%	11, 4.1%	14, 5.2%	
HR (95% CI)	1 (ref)	0.76 (0.32–1.76)	0.86 (0.37–1.98)	0.7210
Cerebrovascular mortality				
Death *n*, %	14, 5.2%	11, 4.1%	6, 2.2%	
HR (95% CI)	1 (ref)	0.79 (0.34–1.78)	0.48 (0.16–1.27)	0.1577

HR—Hazard ratios. ^1^ HR adjusted for age, sex, body mass index, education, smoking status, alcohol intake, physical activity and history of hypertension, dyslipidemia, and diabetes mellitus.

## Supplementary Materials

The following are available online at https://www.mdpi.com/2072-6643/12/4/1052/s1. Table S1: Multivariate-adjusted hazard ratios for cancer mortality according to the tertiles of each food intake. Table S2: Multivariate-adjusted hazard ratios for cardiovascular mortality according to the tertiles of each food intake. Table S3: Multivariate-adjusted hazard ratios for cerebrovascular mortality according to the tertiles of each food intake.
