# Supplementary material for: Dietary Diversity and All-Cause and Cause-Specific Mortality in Japanese Community-Dwelling Older Adults
Source: Nutrients. 2020 Apr 10;12(4):1052. doi: 10.3390/nu12041052 (PMC7230563; doi:10.3390/nu12041052)
Supplement: Supplementary file 1 [file nutrients-12-01052-s001.pdf]

Supplementary Table 1. Multivariate-adjusted hazard ratios[1] for cancer mortality according to tertiles of each food intake

	Tertiles of each food intake			
	Lowest tertile	Middle tertile	Highest tertile	*p*
Cereal				
Median (g/day)	350.0	441.8	603.3	
Number of each tertile	264	268	267	
Death n, %	29, 10.9%	28, 10.4%	35, 13.1%	
HR (95% CI)	1 (ref)	1.01 (0.57-1.79)	1.07 (0.61-1.90)	0.961
Potatoes				
Median (g/day)	15.7	43.8	86.7	
Number of each tertile	261	271	267	
Death n, %	37, 14.1%	30, 11.0%	25, 9.3%	
HR (95% CI)	1 (ref)	0.77 (0.46-1.28)	0.62 (0.35-1.06)	0.215
Beans				
Median (g/day)	25.8	61.7	117	
Number of each tertile	264	264	271	
Death n, %	31, 11.7%	35, 13.2%	26, 9.5%	
HR (95% CI)	1 (ref)	1.09 (0.65-1.84)	0.64 (0.37-1.12)	0.124
Nuts and seeds				
Median (g/day)	0.0	1.3	7.3	
Number of each tertile	265	256	278	
Death n, %	38, 14.3%	31, 12.1%	23, 8.2%	
HR (95% CI)	1 (ref)	0.87 (0.52-1.45)	0.58 (0.33-1.00)	0.145
Non-green yellow vegetables				
Median (g/day)	99.8	172.7	259.1	
Number of each tertile	265	267	267	
Death n, %	36, 13.5%	23, 8.6%	33, 12.3%	
HR (95% CI)	1 (ref)	0.66 (0.37-1.14)	1.13 (0.64-1.97)	0.156
Green yellow vegetables				
Median (g/day)	57.0	114.4	187.6	
Number of each tertile	265	267	267	
Death n, %	31, 11.7%	29, 10.8%	32, 11.9%	
HR (95% CI)	1 (ref)	1.08 (0.61-1.93)	1.38 (0.78-2.48)	0.510
Fruits				
Median (g/day)	61.0	148.5	276.7	
Number of each tertile	262	270	267	
Death n, %	39, 14.8%	30, 11.1%	23, 8.6%	
HR (95% CI)	1 (ref)	0.87 (0.51-1.47)	0.70 (0.39-1.23)	0.470
Mushrooms				
Median (g/day)	0.0	10	27.6	
Number of each tertile	259	271	269	
Death n, %	33, 12.7%	31, 11.4%	28, 10.4%	
HR (95% CI)	1 (ref)	0.90 (0.53-1.54)	0.82 (0.47-1.41)	0.773
Seaweed				
Median (g/day)	3.4	11.3	27.5	
Number of each tertile	265	267	267	
Death n, %	33, 12.4%	22, 8.2%	37, 13.8%	
HR (95% CI)	1 (ref)	0.65 (0.36-1.15)	1.02 (0.61-1.72)	0.222
Fish and shellfish				
Median (g/day)	54.1	89.5	143.3	
Number of each tertile	265	267	267	
Death n, %	27, 10.1%	26, 9.7%	39, 14.6%	
HR (95% CI)	1 (ref)	0.99 (0.56-1.75)	1.32 (0.77-2.28)	0.481
Meats				
Median (g/day)	21.7	48.3	80	
Number of each tertile	265	266	268	
Death n, %	34, 12.8%	31, 11.6%	27, 10.0%	
HR (95% CI)	1 (ref)	1.08 (0.64-1.84)	0.80 (0.45-1.39)	0.538
Eggs				
Median (g/day)	20.0	45	68.8	
Number of each tertile	264	267	268	
Death n, %	30, 11.3%	33, 12.3%	29, 10.8%	
HR (95% CI)	1 (ref)	1.13 (0.68-1.90)	0.82 (0.47-1.42)	0.504
Milk and dairy products				
Median (g/day)	13.3	155.7	285.3	
Number of each tertile	262	270	267	
Death n, %	31, 11.8%	30, 11.1%	31, 11.6%	
HR (95% CI)	1 (ref)	0.87 (0.50-1.49)	1.18 (0.67-2.08)	0.542

HR—Hazard ratio, CI—Confidence Interval, ref—reference.

[1] HR adjusted for age, sex, body mass index, education, smoking status, alcohol intake, physical activity and history of hypertension, dyslipidemia, and diabetes mellitus. All food intakes were entered in a single model.

Supplementary Table 2. Multivariate-adjusted hazard ratios[1] for cardiovascular mortality according to tertiles of each food intake

	Tertiles of each food intake			
	Lowest tertile	Middle tertile	Highest tertile	*p*
Cereal				
Median (g/day)	350.0	441.8	603.3	
Number of each tertile	264	268	267	
Death n, %	14, 5.3%	10, 3.7%	14, 5.2%	
HR (95% CI)	1 (ref)	0.71 (0.26-1.82)	1.09 (0.46-2.60)	0.621
Potatoes				
Median (g/day)	15.7	43.8	86.7	
Number of each tertile	261	271	267	
Death n, %	9, 3.4%	18, 6.6%	11, 4.1%	
HR (95% CI)	1 (ref)	1.65 (0.70-4.13)	0.91 (0.33-2.50)	0.294
Beans				
Median (g/day)	25.8	61.7	117	
Number of each tertile	264	264	271	
Death n, %	12, 4.5%	9, 3.4%	17, 6.2%	
HR (95% CI)	1 (ref)	0.65 (0.25-1.64)	1.39 (0.60-3.29)	0.253
Nuts and seeds				
Median (g/day)	0.0	1.3	7.3	
Number of each tertile	265	256	278	
Death n, %	14, 5.2%	10, 3.9%	14, 5%	
HR (95% CI)	1 (ref)	0.99 (0.40-2.38)	1.46 (0.63-3.41)	0.589
Non-green yellow vegetables				
Median (g/day)	99.8	172.7	259.1	
Number of each tertile	265	267	267	
Death n, %	18, 6.7%	12, 4.4%	8, 3%	
HR (95% CI)	1 (ref)	0.85 (0.36-1.94)	0.40 (0.14-1.06)	0.191
Green yellow vegetables				
Median (g/day)	57.0	114.4	187.6	
Number of each tertile	265	267	267	
Death n, %	14, 5.2%	12, 4.4%	12, 4.4%	
HR (95% CI)	1 (ref)	0.78 (0.32-1.86)	1.02 (0.40-2.55)	0.800
Fruits				
Median (g/day)	61.0	148.5	276.7	
Number of each tertile	262	270	267	
Death n, %	10, 3.8%	11, 4%	17, 6.3%	
HR (95% CI)	1 (ref)	1.29 (0.50-3.41)	2.16 (0.92-5.36)	0.191
Mushrooms				
Median (g/day)	0.0	10	27.6	
Number of each tertile	259	271	269	
Death n, %	19, 7.3%	7, 2.5%	12, 4.4%	
HR (95% CI)	1 (ref)	0.45 (0.17-1.12)	0.81 (0.36-1.76)	0.254
Seaweed				
Median (g/day)	3.4	11.3	27.5	
Number of each tertile	265	267	267	
Death n, %	14, 5.2%	16, 5.9%	8, 3%	
HR (95% CI)	1 (ref)	1.15 (0.51-2.61)	0.64 (0.24-1.60)	0.452
Fish and shellfish				
Median (g/day)	54.1	89.5	143.3	
Number of each tertile	265	267	267	
Death n, %	17, 6.4%	12, 4.4%	9, 3.3%	
HR (95% CI)	1 (ref)	1.17 (0.50-2.74)	0.74 (0.29-1.76)	0.620
Meats				
Median (g/day)	21.7	48.3	80	
Number of each tertile	265	266	268	
Death n, %	12, 4.5%	12, 4.5%	14, 5.2%	
HR (95% CI)	1 (ref)	1.07 (0.43-2.69)	1.71 (0.71-4.17)	0.416
Eggs				
Median (g/day)	20.0	45	68.8	
Number of each tertile	264	267	268	
Death n, %	11, 4.1%	14, 5.2%	13, 4.8%	
HR (95% CI)	1 (ref)	1.02 (0.41-2.52)	1.48 (0.62-3.62)	0.599
Milk and dairy products				
Median (g/day)	13.3	155.7	285.3	
Number of each tertile	262	270	267	
Death n, %	15, 5.7%	11, 4.0%	12, 4.4%	
HR (95% CI)	1 (ref)	0.64 (0.26-1.53)	0.52 (0.20-1.30)	0.359

HR—Hazard ratio, CI—Confidence Interval, ref—reference.

[1] HR adjusted for age, sex, body mass index, education, smoking status, alcohol intake, physical activity and history of hypertension, dyslipidemia, and diabetes mellitus. All food intakes were entered in a single model.

Supplementary Table 3. Multivariate-adjusted hazard ratios[1] for cerebrovascular mortality according to tertiles of each food intake

	Tertiles of each food intake			
	Lowest tertile	Middle tertile	Highest tertile	*p*
Cereal				
Median (g/day)	350.0	441.8	603.3	
Number of each tertile	264	268	267	
Death n, %	12, 4.5%	6, 2.2%	13, 4.8%	
HR (95% CI)	1 (ref)	0.47 (0.15-1.34)	1.11 (0.43-2.90)	0.232
Potatoes				
Median (g/day)	15.7	43.8	86.7	
Number of each tertile	261	271	267	
Death n, %	9, 3.4%	14, 5.1%	8, 3.0%	
HR (95% CI)	1 (ref)	1.29 (0.53-3.28)	0.88 (0.31-2.49)	0.711
Beans				
Median (g/day)	25.8	61.7	117	
Number of each tertile	264	264	271	
Death n, %	11, 4.1%	14, 5.3%	6, 2.2%	
HR (95% CI)	1 (ref)	1.67 (0.67-4.32)	0.60 (0.19-1.73)	0.142
Nuts and seeds				
Median (g/day)	0.0	1.3	7.3	
Number of each tertile	265	256	278	
Death n, %	14, 5.2%	6, 2.3%	11, 3.9%	
HR (95% CI)	1 (ref)	0.48 (0.16-1.31)	0.82 (0.33-2.03)	0.387
Non-green yellow vegetables				
Median (g/day)	99.8	172.7	259.1	
Number of each tertile	265	267	267	
Death n, %	7, 2.6%	14, 5.2%	10, 3.7%	
HR (95% CI)	1 (ref)	2.45 (0.97-6.73)	2.26 (0.77-6.96)	0.167
Green yellow vegetables				
Median (g/day)	57.0	114.4	187.6	
Number of each tertile	265	267	267	
Death n, %	9, 3.4%	12, 4.4%	10, 3.7%	
HR (95% CI)	1 (ref)	1.50 (0.60-3.94)	1.44 (0.54-3.92)	0.663
Fruits				
Median (g/day)	61.0	148.5	276.7	
Number of each tertile	262	270	267	
Death n, %	14, 5.3%	8, 2.9%	9, 3.3%	
HR (95% CI)	1 (ref)	0.63 (0.23-1.62)	0.73 (0.27-1.85)	0.617
Mushrooms				
Median (g/day)	0.0	10	27.6	
Number of each tertile	259	271	269	
Death n, %	11, 4.2%	14, 5.1%	6, 2.2%	
HR (95% CI)	1 (ref)	1.03 (0.43-2.52)	0.54 (0.17-1.51)	0.404
Seaweed				
Median (g/day)	3.4	11.3	27.5	
Number of each tertile	265	267	267	
Death n, %	9, 3.4%	10, 3.7%	12, 4.4%	
HR (95% CI)	1 (ref)	0.80 (0.29-2.20)	1.35 (0.51-3.66)	0.541
Fish and shellfish				
Median (g/day)	54.1	89.5	143.3	
Number of each tertile	265	267	267	
Death n, %	11, 4.1%	9, 3.3%	11, 4.1%	
HR (95% CI)	1 (ref)	0.75 (0.27-2.01)	1.21 (0.46-3.23)	0.634
Meats				
Median (g/day)	21.7	48.3	80	
Number of each tertile	265	266	268	
Death n, %	12, 4.5%	11, 4.1%	8, 2.9%	
HR (95% CI)	1 (ref)	1.07 (0.42-2.73)	1.02 (0.36-2.81)	0.990
Eggs				
Median (g/day)	20.0	45	68.8	
Number of each tertile	264	267	268	
Death n, %	11, 4.1%	8, 3.0%	12, 4.4%	
HR (95% CI)	1 (ref)	0.83 (0.31-2.15)	1.54 (0.64-3.74)	0.402
Milk and dairy products				
Median (g/day)	13.3	155.7	285.3	
Number of each tertile	262	270	267	
Death n, %	15, 5.7%	8, 2.9%	8, 3.0%	
HR (95% CI)	1 (ref)	0.48 (0.17-1.24)	0.56 (0.20-1.49)	0.283

HR—Hazard ratio, CI—Confidence Interval, ref—reference.

[1] HR adjusted for age, sex, body mass index, education, smoking status, alcohol intake, physical activity and history of hypertension, dyslipidemia, and diabetes mellitus. All food intakes were entered in a single model.
